# Sarcosine promotes trafficking of dendritic cells and improves efficacy of anti-tumor dendritic cell vaccines via CXC chemokine family signaling

**DOI:** 10.1186/s40425-019-0809-4

**Published:** 2019-11-21

**Authors:** Farhad Dastmalchi, Aida Karachi, Changlin Yang, Hassan Azari, Elias Joseph Sayour, Anjelika Dechkovskaia, Alexander Loren Vlasak, Megan Ellen Saia, Rolando Eladio Lovaton, Duane Anthony Mitchell, Maryam Rahman

**Affiliations:** 1 Preston A. Wells, Jr. Center for Brain Tumor Therapy, UF Brain Tumor Immunotherapy Program, University of Florida, Gainesville, FL USA; 2 Neurosurgery Service, Hospital Cayetano Heredia, Lima, Peru

**Keywords:** Dendritic cell vaccine, Immunotherapy, Glioblastoma, Cell migration, Sarcosine, CXCR2

## Abstract

**Background:**

Dendritic cell (DC) vaccine efficacy is directly related to the efficiency of DC migration to the lymph node after delivery to the patient. We discovered that a naturally occurring metabolite, sarcosine, increases DC migration in human and murine cells resulting in significantly improved anti-tumor efficacy. We hypothesized that sarcosine induced cell migration was due to chemokine signaling.

**Methods:**

DCs were harvested from the bone marrow of wild type C57BL/6 mice and electroporated with tumor messenger RNA (mRNA). Human DCs were isolated from peripheral blood mononuclear cells (PBMCs). DCs were treated with 20 mM of sarcosine. Antigen specific T cells were isolated from transgenic mice and injected intravenously into tumor bearing mice. DC vaccines were delivered via intradermal injection. In vivo migration was evaluated by flow cytometry and immunofluorescence microscopy. Gene expression in RNA was investigated in DCs via RT-PCR and Nanostring.

**Results:**

Sarcosine significantly increased human and murine DC migration in vitro. In vivo sarcosine-treated DCs had significantly increased migration to both the lymph nodes and spleens after intradermal delivery in mice. Sarcosine-treated DC vaccines resulted in significantly improved tumor control in a B16F10-OVA tumor flank model and improved survival in an intracranial GL261-gp100 glioma model. Gene expression demonstrated an upregulation of CXCR2, CXCL3 and CXCL1 in sarcosine- treated DCs. Further metabolic analysis demonstrated the up-regulation of cyclooxygenase-1 and Pik3cg. Sarcosine induced migration was abrogated by adding the CXCR2 neutralizing antibody in both human and murine DCs. CXCR2 neutralizing antibody also removed the survival benefit of sarcosine-treated DCs in the tumor models.

**Conclusion:**

Sarcosine increases the migration of murine and human DCs via the CXC chemokine pathway. This platform can be utilized to improve existing DC vaccine strategies.

## Background

Antigen presenting cells (APCs) such as dendritic cells (DCs) play a critical role in activating the adaptive immune response against pathogens. DCs can effectively stimulate T cells via specialized pathways and activate them against specific antigens including antigen relevant in patients with cancer. This mechanism leads to potent immune responses that can be leveraged for the treatment of traditionally resistant tumors such as glioblastoma. DC vaccines are a novel and versatile treatment approach, and this strategy is already FDA approved for the treatment of prostate cancer [1, 2].

Due to the versatility of DC vaccines, studies are ongoing for their use in resistant malignancies like glioblastoma (GBM). Phase I/II trials in GBM patients demonstrate feasibility and safety of creating and delivering DC vaccines as well as robust anti-tumor immune responses in select patients [1, 3–5]. Importantly, DC vaccine efficacy has been shown to be tightly related to the efficiency of DC migration to the lymph node after delivery to the patient [6]. Our group has demonstrated that patients with GBM who had increased DC migration by co-vaccinating with tetanus toxoid had significantly larger anti-tumor immune responses and improved survival (18.5 versus 36.6 months respectively in the control and treatment arms) [6]. Therefore, methods to improve immune cell migration have the potential to make cellular immunotherapeutic strategies more potent.

In the search for a metabolite to track DCs in vivo, we began experimenting with sarcosine (N-methyl glycine) to label DCs for tracking the cells in vivo using MRI. We chose sarcosine because it is naturally occurring and non-toxic, has low expression in brain and lymph node tissue, and it is commercially available. During experimentation, we observed that sarcosine treated DCs seemed to have improved migration. We therefore began exploring the use of sarcosine as an adjuvant during DC vaccination and its impact on anti-tumor efficacy. The objective of these studies was to characterize the impact of sarcosine on DC function and migration in the context of an intradermal DC vaccine for the treatment of intracranial tumor models.

## Methods

### Sarcosine assay

Sarcosine 98% (Synonym: N-Methylglycine) was purchased from Sigma-Aldrich. Sarcosine was dissolved in T cell or DC culture medium at 20 mM and sterile solution was prepared via passing through 0.22 um low protein binding Durapore membrane (Life Science). Intra cellular sarcosine concentration was evaluated with sarcosine assay kit from Sigma-Aldrich that detects the concentration of sarcosine via colorimetric observation at 570 nm. DCs were collected from culture media in different groups and were mixed with sarcosine assay buffer, sarcosine probe and sarcosine enzyme mix that were provided in the kit. Sarcosine standard samples were prepared. The manufacturer instructions were followed and samples were incubated in plates in a dark environment at 37 °C for 60 min. Concentrations of sarcosine in different groups were measured via colorimetric method.

### DC vaccine generation

Bone marrow (BM) was harvested from the long bones and sternum of C57BL/6 mice which were purchased from Jackson Laboratory (Bar Harbor, ME). All animal projects and protocols were initiated after achieving the approval from the Institutional Animal Care and Use Committee (IACUC) of the University of Florida. Myeloid-derived cells were cultured in DC complete media including granulocyte macrophage colony stimulating factor (GM-CSF) and interleukin 4 (IL-4). Cells were cultured in six well plates for 6 days. At day 7, cells were re-plated in 60 mm dishes and DCs were electroporated with OVA-mRNA at day 8. At day 9, DCs were collected in phosphate buffered saline (PBS) for administration. DC vaccines were delivered via intradermal injection in the inguinal area.

### Human DC vaccine generation

Whole blood from five healthy donors was purchased from Life South blood bank. Human DCs were generated from blood monocytes by incubating them with IL-4, GM-CSF, TNF-α, IL-1β and IL-6. This method has been previously described [7]. pp65 RNA was produced and transfected from the full-length cDNA that was donated by Dr. Bill Britt (University of Alabama-Birmingham, Birmingham, Alabama).

### Adoptive cell transfer

Spleens were harvested from PMEL or OT-I T transgenic mice. They were sliced and passed through cell strainer. Lysing solution was used to get rid of red blood cells. Supernatant was discarded and splenocytes pellet was re-suspended at 3 × 10^7^ cells/50 ul of PBS for Intravenous (IV) infusion.

### In vitro migration assay

DCs from different groups were transferred to a Corning Costar Transwell plate (Pore Size: 5.0 μm; 6.5 mm Diameter; 0.33cm^2^ Growth Area) that included an upper and lower chamber. The upper chamber had cells in 100 ul media without cytokines and serum. The lower chamber contained 500 ul of media with serum, CCL19 (250 ng/ml), CCL21 (250 ng/ml) and CXCL3 (250 ng/ml). The upper chamber was cast off after 5 h and the migrated cells into the lower chamber were counted on hemocytometer. In the in vitro migration experiments, CXCR2 activity was inhibited by treating cells for one hour before the migration assay with mouse anti-CXCR2 (at 1 to 50 dilution, Clone242216, R&D Systems) [8]. For in vivo application, SB225002 (selective non-peptide antagonist of CXCR2, Sigma-Aldrich) was dissolved in vehicle (NaCl 0.9% solution plus Tween-80 0.33%) according to manufacturer’s instructions. SB225002 was injected into the mice through intraperitoneal (IP) at 50 μg (1.4 × 10–7 mol) in 200 μl per animal one hour prior each DC vaccine injection [9, 10]. In the human DC in vitro migration experiments, cells were treated with SB225002 at a concentration of 10 μM for one hour prior to the migration studies [11].

### Tumor model

For the intracranial tumor model, GL261-Gp100 tumor cell suspension was prepared at 2 × 10^5^ cells in 1:1 mixture of PBS and methylcellulose in total volume of 2.5 ul per brain. Tumor cells were implanted into the intracranial space stereotactically. The injection needle was positioned 2 mm to right and 1–3 mm above the sagittal-bregma intersection. Once the needle was inserted 3–4 mm into the brain, 2.5 ul of prepared cell mixture was injected over one minute. Bone was used to cover the injection site. One day after tumor implantation, the first GP100-RNA pulsed DC vaccine (1 × 10^6^ cells/mouse) was injected intradermally and 48 h later 3 × 10^7^ PMEL splenocytes were given IV. Then treatment groups received second and third GP100-RNA pulsed DC vaccines every 5 days intradermally. Animals were followed for survival analysis and were euthanized when they reached endpoint.

For the flank model, B16F10-OVA cells were grown in Dulbeccos modified eagles medium (DMEM) and the cells were inoculated into the flank of C57BL/6 mice subcutaneously at a concentration of 1 × 10^6^ cells per 100 ml PBS. The tumor bearing mice were randomized before the first DC vaccine injection. At day 8 of post tumor implantation, 3 × 10^7^ OT-I splenocytes were given IV and the first OVA-RNA pulsed DC vaccine was injected intradermal (1 × 10^6^ cells/mouse). At day 10 post tumor implantation tumor size was measured at flank sites every 2 days. Mice received a second and third vaccine of OVA-RNA pulsed DC vaccines on day 12 and 16. Tumor volume was calculated in millimeters cubed (mm^3^) by the formula (length x width^2^ × 0.52) in a rectangular fashion. The animals were euthanized when tumor growth passed two centimeter in any dimensions or ulceration occurred in tumor side. A linear model was accounted to analyze the correlation of tumor volume and time in each animal.

### Immunofluorescence microscopy

DCs were stained with PKH26 (Red Fluorescent Cell membrane stain, Sigma-Aldrich) immediately before intradermal injection in the inguinal area. At 48 h post DC vaccine administration, spleens or inguinal lymph nodes were collected and immediately embedded inside optimal cutting temperature (OCT) compound then frozen inside the chamber that contains liquid nitrogen. The samples were cut via HM 505E cryostat machine at 6 um thickness and transferred on glass slides for immunofluorescence microscopy analysis. The slides washed with PBS at room temperature and non- specific sites were blocked by incubating sections with 2% rabbit serum was used to block the nonspecific sites in the tissue sections. Fluorophore conjugated antibodies (Anti-CD45R/B220 and Streptavidin, eBioscience)were added to the sections and incubated for overnight at 4 °C. The final stained sections were observed via EVOS inverted immunofluorescence microscope at different objectives.

### Flow cytometry

To evaluate the in vivo migration, bone marrow-derived DCs from green fluorescent protein (GFP) expressing transgenic mice were injected into the wild type C57BL/6 mice intradermal. Draining inguinal lymph nodes (LN) were harvested, digested and single cell suspensions were prepared to count GFP expressing cells, and absolute cell counts were quantified in each sample.

For immune response analysis, spleens were harvested from vaccinated mice after 7 days post vaccination. RBCs were lysed, then splenocytes were transferred into in RPMI-1640 medium (Invitrogen) containing with 10% FCS, 1% l-glutamine, 1% penicillin/streptomycin. 2 × 10^6^ cells were transferred to each well in round-bottomed 96-well plates. Cells were centrifuged at 500 g for 5 min. The cells were resuspended in staining buffer (Thermo Fisher Scientific) and stained with different conjugated antibodies at room temperature for 15 min in dark environments. The cells were stained with fixable-yellow Live/dead Then anti-CD3 (APC-Cy7), anti-CD4 (BV421), anti-CD8 (FITC), anti-CD25 (APC) from BD Bioscience and OT-1 tetramer (PE) from MGL and followed by fixation with 2% paraformaldehyde for 5 min at room temperature.

To evaluate cell phenotype, sarcosine treated and untreated DC cells were prepared by using the above method. Then murine DC cells were stained in separate wells with anti-CD11c (PE), anti-MHC class II (PE), anti-CD80 (PE) and anti-CD86 (PE) from BD Bioscience. Human monocyte derived DCs were stained with anti-CD11c (PE), anti-CD86 (PE) and anti-HLA-DR2 (PE) from BD Bioscience. The BD LSR flow cytometer and FlowJo software were used for all flow cytometry analyses.

### Gene expression

RNA was extracted from murine myeloid DCs by RNeasy Mini Kit (QIAGEN). After reverse transcripting the extracted RNA, cDNA was implemented for gene expression analysis. Genomic expression of mouse cytokines, chemokines and chemokine receptors was evaluated using PrimePCR Selections 96 wells array plates (Bio-Rad). PrimePCR Selections 96 well array plates were analyzed using Bio-Rad Reverse Transcription PCR (RT-PCR) machine (CFX96 Touch).

### Nanostring

An nCounter muse myeloid immune cells profiling panel was implicated to analyze the gene expression via NanoString™ Technology (XT_PGX_MmV2_Myeloid_CSO). Total RNA was extracted from sarcosine treated murine myeloid-derived DCs and the control DCs using RNeasy kit (Qiagen). nSolver software was used for data analyzing, and data was normalized by implication of positive, negative control probes and housekeeping genes.

### Detection of intracellular oxidative stress level

To evaluate the intracellular oxidative stress level, sarcosine treated and untreated murine BM-DCs were prepared by using the above method. DCs were collected at day 7 without any electroporation and washed with warm PBS. Cells were reconstituted at 1.5 × 10^5^ cells/well. Then CM-H2DCFDA (General Oxidative Stress Indicator, Invitrogen) dye was added to cells with 10 μM and the cells were incubated at 37 °C for 60 min based on manufacturer protocol. Then cells washed three times with warm PBS and returned to pre-warmed growth medium and incubated at 37°c for 10 min. Cell plates were measured on fluorescence plate reader at Excitation: 495/Emission: 527 nm. For negative control, unstained cells were examined to deduct the autofluorescence. For positive control, cells were treated with tret-butyl hydroperoxide (TBHP) with 100 μM. Fluorescence intensity was evaluated based on Z score. (z = (expression in treatment sample - mean expression in negative control sample) / standard deviation of expression in negative control sample).

### Measurement of antigen uptake

Sarcosine treated and untreated murine BM-DCs generated as described above. Cells were incubated with 2 mg/ml FITC-OVA at 37 °C for 90 min. Then cells were washed three times with PBS and stained with PE-anti-CD11c. FITC-OVA uptake was evaluated as mean fluorescence intensity (MFI) in CD11c + population. Nonspecific signal of FITC was quantified by incubating cells in 2 mg/ml OVA-FITC at 0 °C for 90 min [12].

### Proliferation assay

Spleens were collected from OT-I mice (from Jackson Laboratories) and CD8+ T cells were isolated through magnetic isolation kit (Miltenyi Biotec). CD8+ T cells were reconstituted in 96 well plate at 1 × 10^5^/200 μl/well and labeled with carboxy fluoroscein succinimidyl ester (CFSE; Molecular Probes). Murine BM-DCs were generated and prepared as described above and electroporated with OVA-mRNA. Labeled CD8+ T cells were co-cultured with sarcosine treated and untreated BM-DCs at 2.5 × 10^4^ cells/200 μl/well. Cells were cultured in RPMI 1640 medium with 10% Fetal Bovine Serum (FBS). Non-electroporated BM-DCs were co-cultured with CD8+ T cells as a negative control. After three days cells harvested and T cells proliferation was quantified through flow cytometry by analyzing CFSE dilution in CD3+/CD8+ population.

### Statistical analysis

GraphPad Prism 7 software was used for all statistical analysis. Statistics were analyzed using one-way ANOVA for studies with multiple groups and Mann Whitney or t-tests for comparisons of two groups. Two way ANOVA was used for tumor volume and body weight analysis. Median survival was analyzed using Log-rank (Mantel-Cox) test. Unpaired t-test method was accounted for flow cytometry data. The data was considered statistically significant when *p* value was < 0.05. The level of significance was indicated via asterisks including *p* > 0.05 not significant, *p < 0.05, **p ≤ 0.01 and ***p < 0.001.

## Results

### Loading cells with sarcosine

**Fig. 1:**
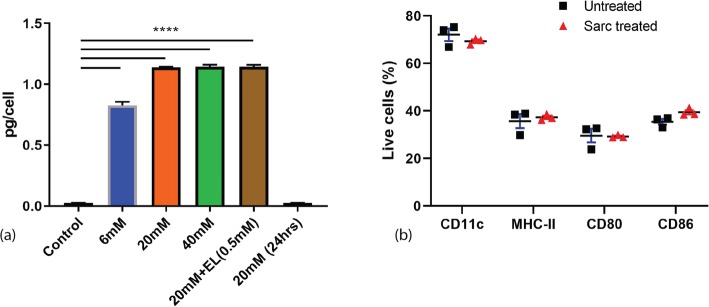
Sarcosine concentration and cell phenotype after treating murine BM-DCs with sarcosine. **a** Mean concentration of intracellular sarcosine. 0.02517pg/cell for control, 0.8274pg/cell for 6mM, 1.14pg/cell for 20mM, 1.145pg/cell for 40mM, 1.145pg/cell for 20mM+EL (0.5mM) and 0.02467pg/cell for 20mM (24hrs) (*p*<0.0001, ANOVA, *n*=12 per group). **b** Flow cytometry of murine BM-DCs to evaluate DC markers CD11c, MHC-II, CD80 and CD86. (BM-DCs collected from 10 mice and experiment was performed in triplicate for each group). BM-DCs=bone marrow derived dendritic cells; DC=dendritic cells; EL=electroporation

To determine the utility of sarcosine in improving DC migration, we first aimed to optimize the intracellular concentration of sarcosine and to investigate its impact on cellular phenotype. DC cells were cultured at various concentrations of sarcosine or electroporated with sarcosine. Cells were collected and a sarcosine was measured using chromometric analysis. Sarcosine concentration within cells increased up to 1.17 pg/cell when cells were cultured at 20 mM of sarcosine. This value did not increase with higher concentrations of sarcosine or electroporation. Additionally, the intracellular sarcosine returned to control levels when cells were removed from sarcosine containing media within 24 h. Therefore, sarcosine could only be increased within the DCs transiently and electroporation with sarcosine was not necessary (Fig. 1a).

The impact of sarcosine on cell phenotype was evaluated using flow cytometry. All groups were cultured in DC media that contained sarcosine for 48 h. Sarcosine treatment did not result in cell death or changes in CD11c, MHC-II, CD80 and CD86 expression compared to control DCs (Fig. 1b).

### In vitro and in vivo migration

**Fig. 2:**
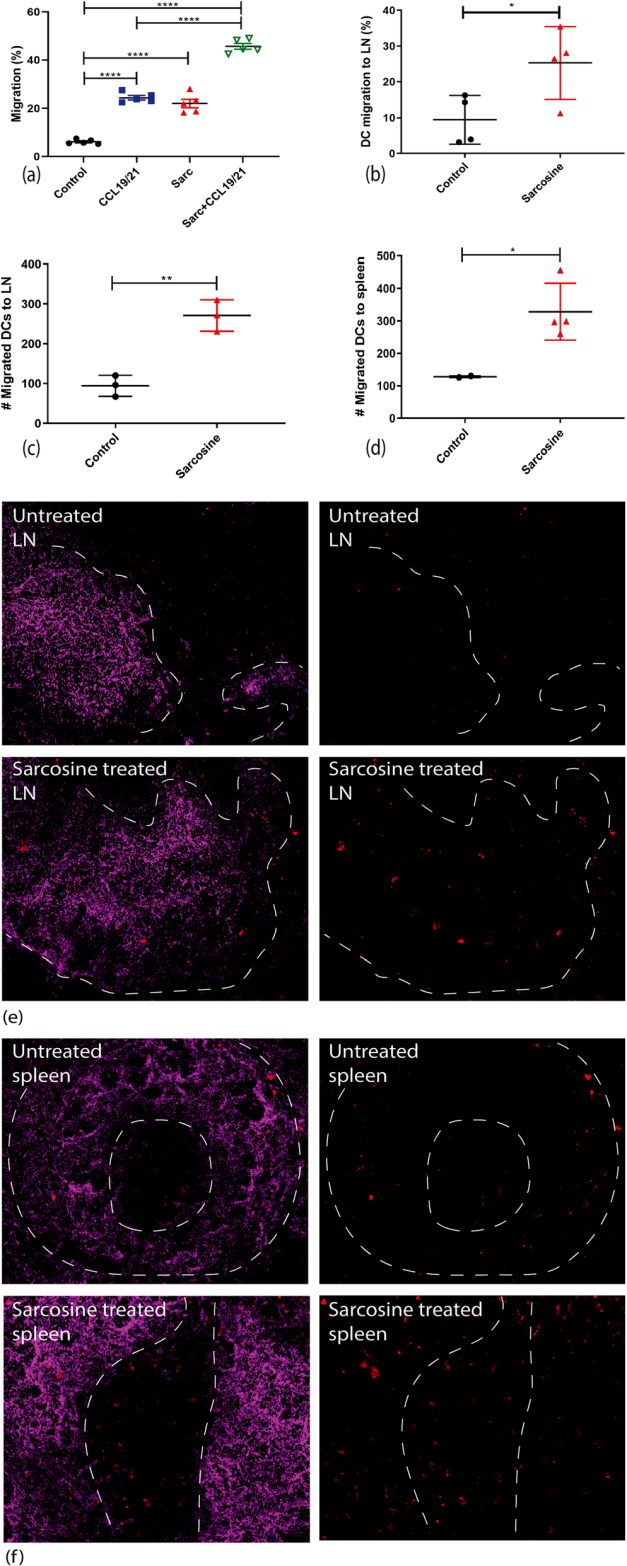
In vitro and in vivo migration assays with sarcosine treated Murine BM-DCs. **a** Sarcosine treated-DCs tested in trans-well in vitro migration analysis revealed that DCs had increased migration with CCL19/21 alone (mean 24.45%) or sarcosine alone (mean 22.05%) compared to control group (mean 6.150%). When DCs were treated with sarcosine and chemokines migration was further enhanced (mean 45.70%, *p* <0.0001, one-way ANOVA). Murine BM-DCs collected from 10 mice for each group and experiment was repeated five times. **b** Migrated DCs to draining LN evaluated by flow cytometry after 48 hours post injection. The mean percent migration was 9.457% for control and 25.30% for sarcosine treated DCs (*p* < 0.0411, unpaired t test) (*n*=4). **c** Migrated-PKH labeled DCs from LN evaluated by immunofluorescent microscopy after 48 hours. Mean was 94.33 cells for control and 271.0 cells for sarcosine treated DCs (*p* < 0.0030, unpaired t test) (*n*=3). **d** Migrated-PKH dye labeled DCs from spleen evaluated by immunofluorescent microscopy after 48 hours. Mean was 128.0 cells for control and 328.5 cells for sarcosine treated DCs (*p* < 0.0378, unpaired t test) (*n*=4). **e** Immunofluorescent microscopy observation from draining lymph node 48 hours post vaccination. **f** Immunofluorescent microscopy observation from spleen 48 hours post vaccination. Purple=B220, Red=PKH labeled DCs. White dotted lines were used to indicate the margin between white pulp from red pulp. LN=lymph node; BM-DCs=bone marrow derived dendritic cells; DCs=dendritic cells

Sarcosine effects on DC migration were tested using a trans-well migration assay that uses chemokines to assess DC migration in vitro. The chamber was loaded with CCL19/21 on one side and DCs were loaded to assess migration towards the chemokines. DCs were treated with sarcosine at 20 mM for 24 h and electroporated with OVA-RNA for DC maturation. DCs had increased migration with CCL19/21 alone (mean 24.45%) or sarcosine alone (mean 22.05%) compared to the control group. When DCs were treated with sarcosine and chemokines were added to the other chamber, DCs migrated even more efficiently than either sarcosine loading alone or chemokines alone (mean 45.70%, *p* < 0.0001) (Fig. 2a).

Next, bone marrow derived DCs were used to test in vivo migration. C57BL/6 mice received the DC vaccines via intradermal injection in the inguinal area. Forty-eight hours post vaccination, draining lymph nodes and spleens were harvested for flow cytometry and immunofluorescence microscopy. Sarcosine treated DCs had significantly increased migration to the draining lymph nodes measured by flow cytometry (mean 9.457% cells for control versus 25.30% for sarcosine treated DCs, *p* < 0.0411) (Fig. 2b). This was confirmed using immunofluorescence (Fig. 2c, e). Interestingly, these DCs were also visualized in the spleens at just 48 h after intradermal injection (328.5 ± 43.71 control versus 128 ± 2 cells sarcosine, *p* < 0.05) (Fig. 2d & f).

Other functions of DCs were also tested in the setting of sarcosine treatment. Antigen uptake was measured in sarcosine treated DCs by co-culturing cells with FITC-OVA. Sarcosine resulted in decreased antigen uptake (Additional file 1: Figure S1a) However, antigen presentation measured using a T cell proliferation assay was not affected by sarcosine treatment (Additional file 1: Figure S1b-e).

### Systemic immune response and anti-tumor efficacy of sarcosine treated DCs

**Fig. 3:**
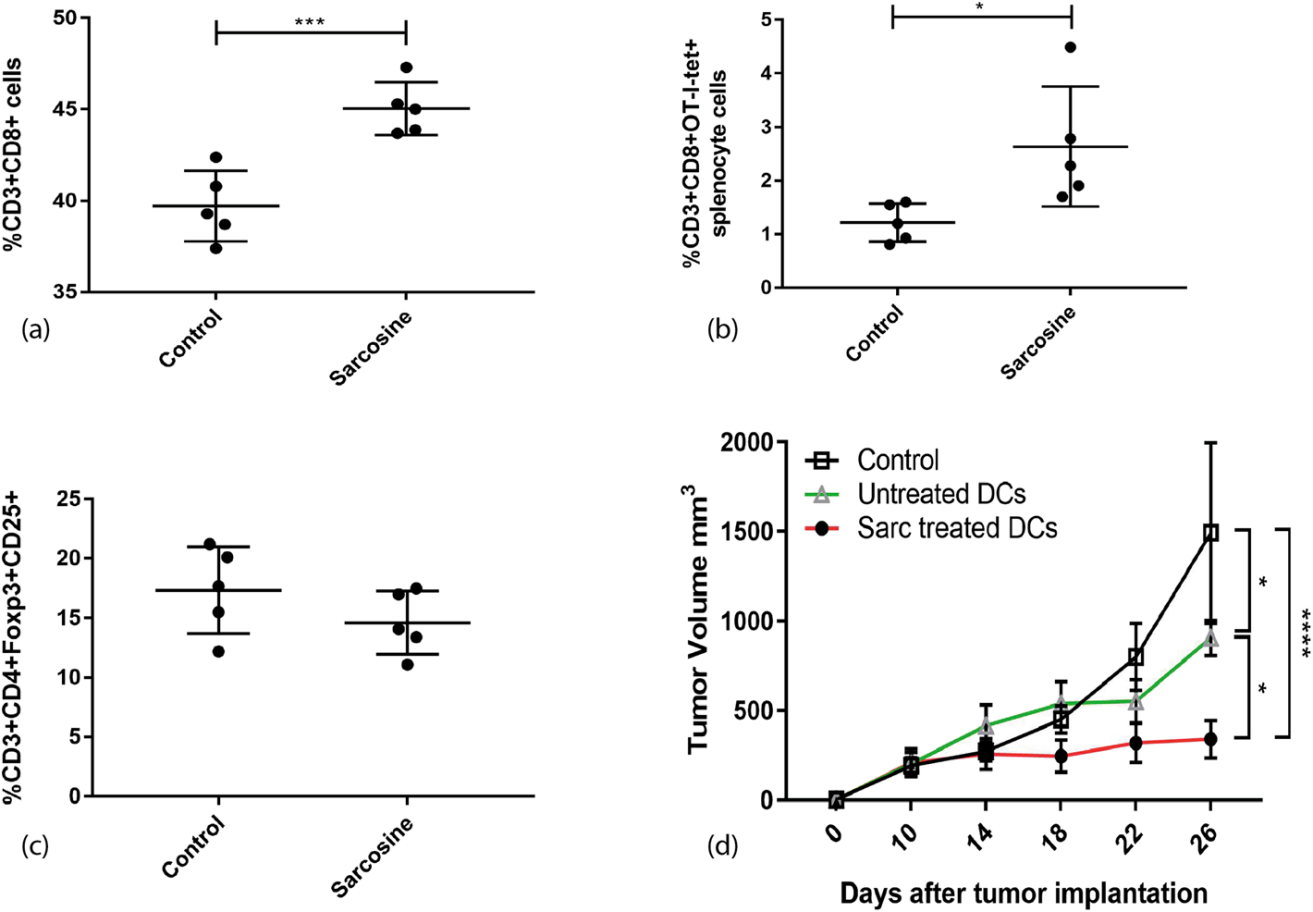
The immunological response of sarcosine treated DCs in non-tumor bearing mice and the efficacy of sarcosine treated DCs in tumor bearing mice. **a** Splenocytes were analyzed by flow cytometry after tumor bearing mice received DC vaccines. CD8 T cells increased in mice treated with sarcosine treated DCs. Mean 39.72% ± 0.86 for control and 45.04% ± 0.64 for sarcosine treated DCs. (*p* < 0.0011, unpaired t test, *n*=5). **b** Antigen specific CD8 T cells in the spleens also increased in sarcosine treated DC group. Mean 1.22 ± 0.16% for control and 2.63 ± 0.50% for sarcosine treated DCs. (*p* < 0.0270, unpaired t test, *n*=5). **c** Tregs were similar between groups. Mean 17.34% ± 1.62 for control and 14.62% ± 1.19 for sarcosine treated DCs. (*p* < 0.2124, unpaired t test, *n*=5). **d** B16F10-OVA tumor bearing animals were treated with sarcosine treated DCs versus control DCs. Sarcosine treated DCs reduced tumor growth significantly. Mean tumor volume 1491 mm^3^ for control, 905 mm^3^ for DC and 338.8 mm^3^ for sarcosine treated DCs at day 26. (*p*=<0.0001, 2 way ANOVA, *n*=10)

The increase in DC migration to the lymph nodes has previously been shown to increase the adaptive immune response [6]. To test if the sarcosine treated DCs increased T cell proliferation, non-tumor bearing mice underwent infusion with OT-1 T cells followed by DC vaccination with untreated OVA-RNA pulsed DCs or sarcosine treated OVA-RNA pulsed DCs. Vaccination with sarcosine treated DCs increased the percent of CD8 T cells compared to animals treated with control DC vaccination (45.04% ± 0.6431 versus 39.72% ± 0.8645, *n* = 5, *p* < 0.0011) (Fig. 3a). Additionally, an increase in percent of antigen specific OT-1 T cells was observed (2.634% ± 0.4995 versus 1.218% ± 0.159, n = 5, *p* < 0.0270) (Fig. 3b). The percent of Tregs was unchanged between control and sarcosine treated DC groups (Fig. 3c).

The B16F10-OVA melanoma flank model was used to test the impact of sarcosine DCs on tumor growth. After tumor implantation, animals received infusion of antigen specific OT-1 splenocytes and DC vaccines with or without sarcosine 10 days post tumor injection. The animals that treated with sarcosine treated DCs had significantly slowed tumor growth over time compared to animals receiving non-sarcosine treated DC vaccines. Twenty-six days after tumor implantation the mean tumor volume was 1491 mm^3^ for the controls, 905 mm^3^ for DC treated mice, and 338.8 mm^3^ for sarcosine treated DC treated mice (p = < 0.0001, 2 way ANOVA, *n* = 10) (Fig. 3d). Sarcosine treated DC treatment did not result in toxicity as measured by total body weight (Additional file 1: Figure S2a). Sarcosine treated B16F10-OVA tumor cells were also not associated with an increase in tumor growth or invasion (Additional file 1: Figure S2b).

### Gene expression analysis of sarcosine treated DCs

**Fig. 4:**
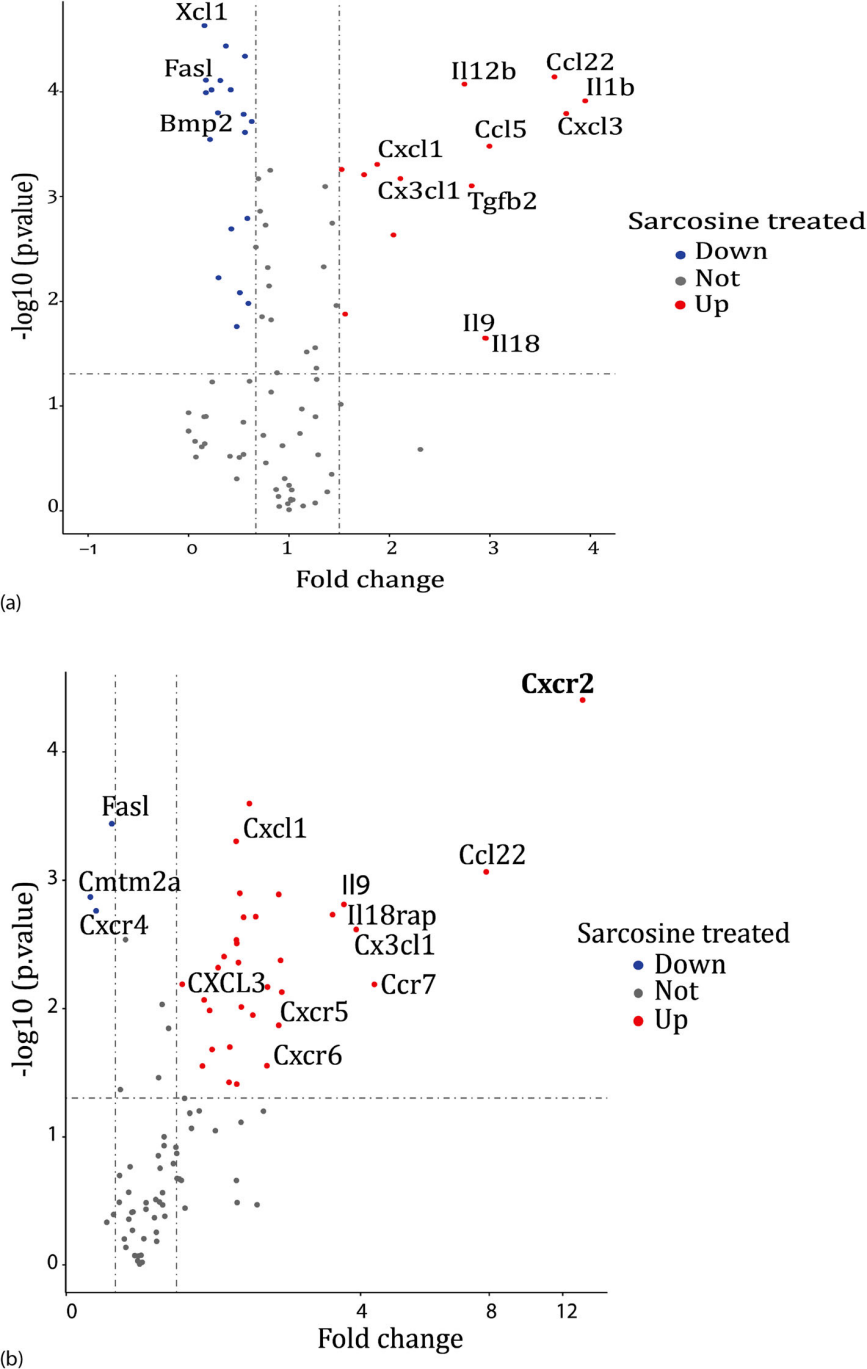
Gene expression analysis of sarcosine treated DCs. (a) Sarcosine treated murine BM-DCs tested for cytokine and chemokine expression (*p* value< 0.05, Volcano R-plot, *n*=3). (b) Sarcosine treated murine myeloid-derived DCs tested for cytokine and chemokine receptors (*p* value< 0.05, Volcano R-plot, *n*=3)

The genomic expression was analyzed by isolating RNA extracted from murine sarcosine-treated DCs to further understand the mechanism of sarcosine induced cell migration. Cytokine and chemokine receptor gene expression was analyzed using RT-PCR. These analyses demonstrated sarcosine upregulated CCL22, CXCL3, IL1b, IL12b, CCL5, and CXCL1 and downregulated XCL1, FASL, and BMP2 (Fig. 4a). We then tested for chemokine receptors and found several significant upregulations including CXCR2 and CCL22, CXCL3, CX3CL1, CXCR5, IL9, IL18rap and CCR7. Downregulated receptors included FASL, CMTM2a, CXCR4 in sarcosine treated DCs (Fig. 4b).

**Fig. 5:**
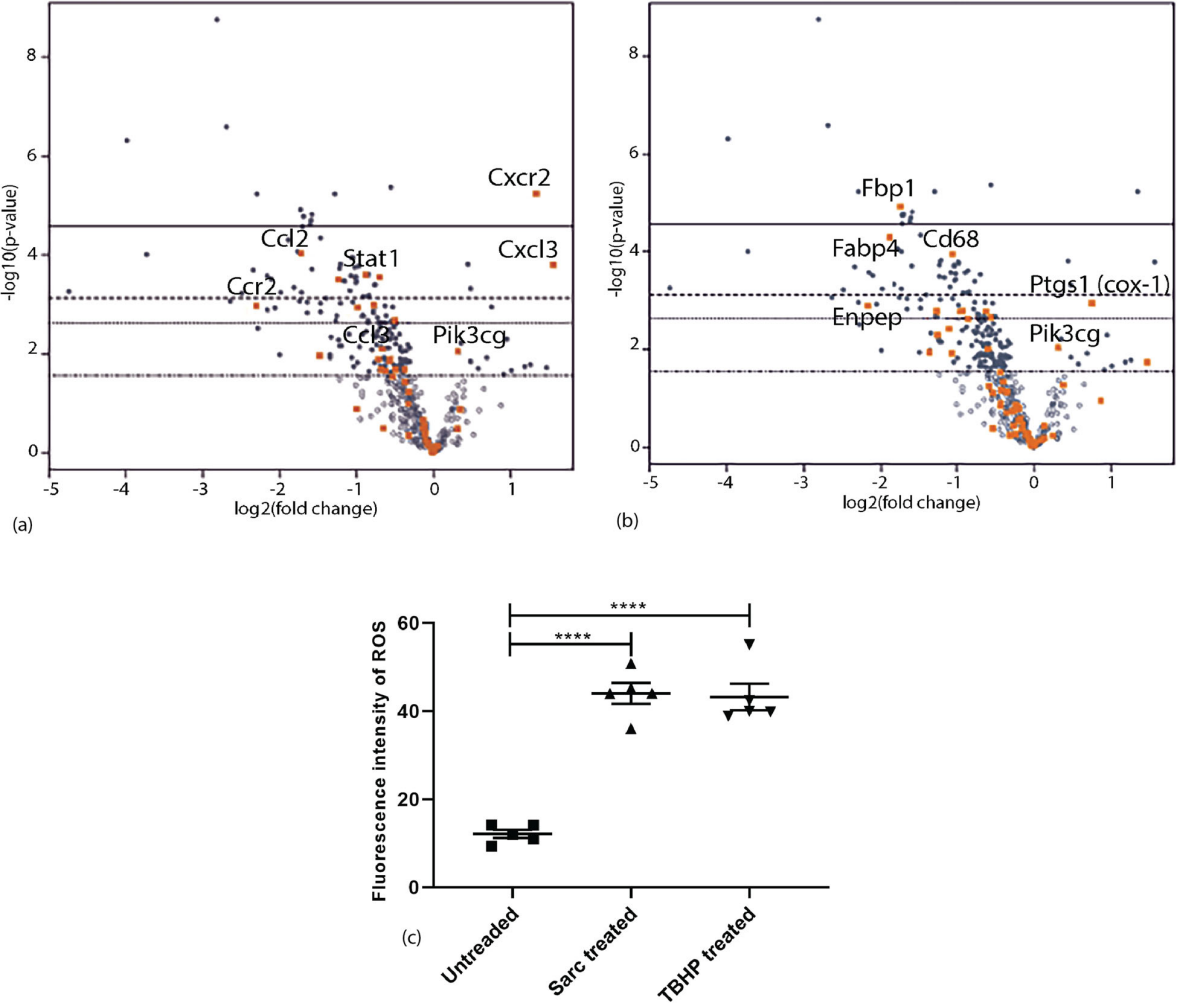
Sarcosine and cellular metabolism. **a** Nanostring ™ analysis of murine myeloid-derived DCs treated with sarcosine were compared to control DCs. CXCR2 (*p*<0.01) and CXCL3 (*p*<0.05) were highly expressed in the sarcosine-treated group. **b** Nanostring metabolic pathway analysis demonstrated overexpression of Ptgs1 (Cyclooxygenase 1) (*p*<0.1) and Pik3cg (*p*<0.5) genes. **c** The presence of reactive oxidative species (ROS) was evaluated based on fluorescence intensity in murine BM-DC. Sarcosine increased ROS in BM-DCs compare to untreated BM-DCs. Mean fluorescence intensity was 12.14 for untreated DCs, 44.05 for sarcosine treated DCs and 43.23 for positive control (*p* <0.0001, one-way ANOVA, *n*=5)

To further analyze the mechanism by which sarcosine was increasing DC migration, RNA was isolated from bone marrow derived DCs after being cultured in sarcosine. Nanostring™ analysis was performed. Sarcosine treated DCs had a significant upregulation of CXCR2 (*p* < 0.01) and CXCL3 (*p* < 0.05) (Fig. 5a). Further metabolic analysis showed that cyclooxygenase 1 (Cox 1) and Pik3cg were also upregulated (Fig. 5b). Based on these findings combined with a lack of upregulation in other key pathways, we hypothesized that the mechanism of sarcosine action was through a decrease in glycine that leads to intracellular oxidative stress and resultant increase of Cox 1 leading to an upregulation of CXC chemokine signaling. Intracellular oxidative stress was evaluated and sarcosine increased the presence of reactive oxidative species (ROS) in DCs compare to untreated DCs (mean 12.14 fluorescence intensity (FI) for untreated DCs vs. 44.05 FI for sarcosine treated DCs, *p* < 0.0001, one-way ANOVA) (Fig. 5c).

### CXCR2 blockade effect on sarcosine induced migration

**Fig. 6:**
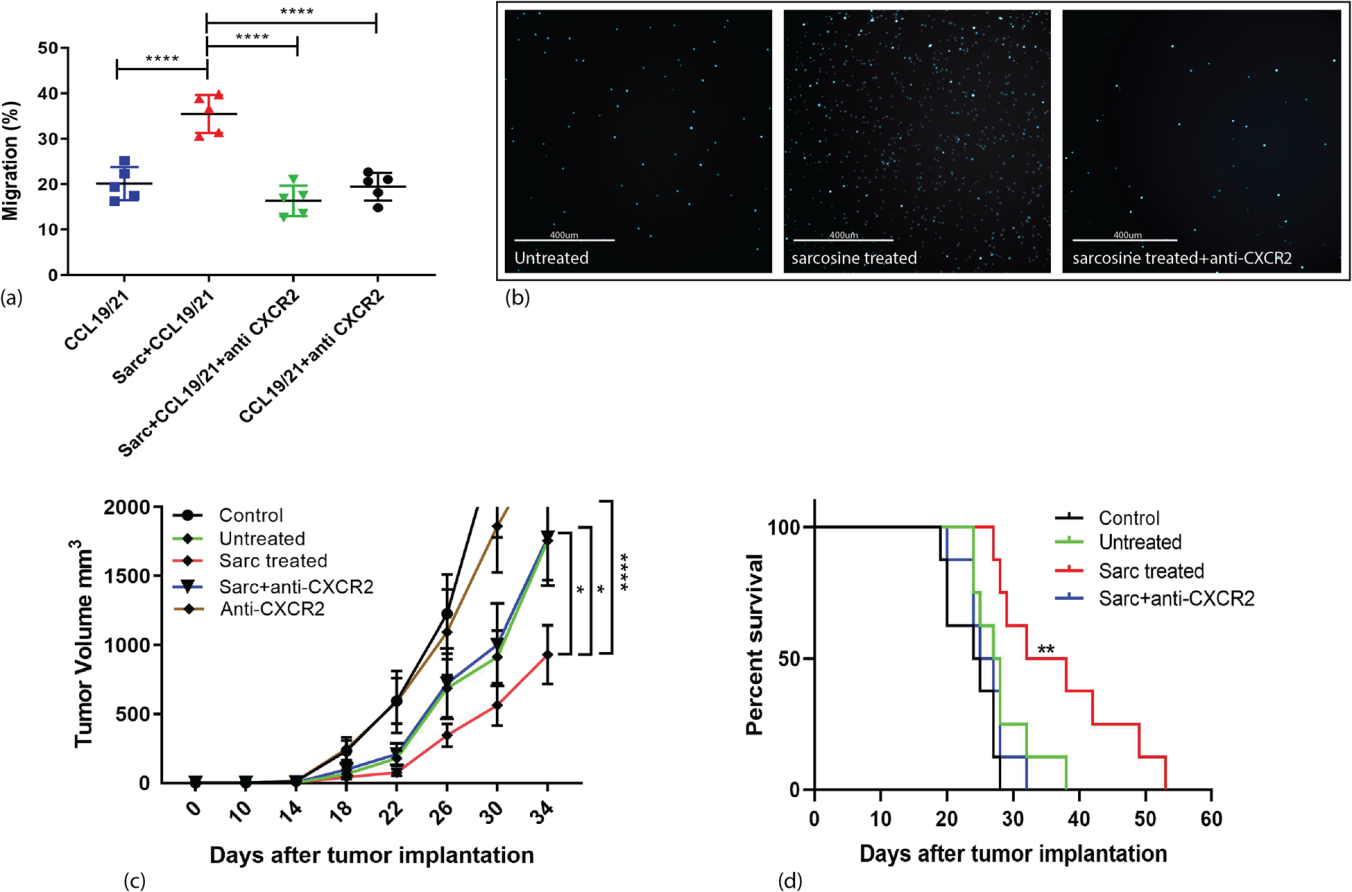
Analysis of murine DCs treated with sarcosine in presence of CXCR2 neutralizing antibody. **a** In vitro trans-well migration analysis of sarcosine treated DCs cultured with CXCR2 neutralizing antibody. Mean percentage of migrated cells were 20.13% for CCL19/21 alone, 35.5% for sarcosine+CCL19/21, 16.33% for sarcosine+CCL19/21+anti-CXCR2, and 19.46% for CCL19/21+anti-CXCR2 with CCL19/21. (*p* < 0.0001, one-way ANOVA, *n*=5). **b** Immunofluorescence microscopy observation of trans-well migration of sarcosine treated DCs cultured with CXCR2 neutralizing antibody. **c** B16F10-OVA tumor bearing animals were treated with sarcosine treated DCs versus non-sarcosine treated DCs in presence of CXCR2 neutralizing antibody. Mean difference in tumor volume was 1756 mm^3^ control vs. DCs, 1996 mm^3^ for control vs. sarcosine treated DCs and 825.4 mm^3^ for DCs vs. sarcosine treated DCs at day 34 (*p*=<0.0001, 2 way ANOVA, *n*=8). **d** GL261-GP100 tumor bearing animals were treated DCs with or without CXCR2 neutralizing antibody. Kaplan-Meier survival analysis showed that sarcosine significantly prolonged survival from naive DC vaccine treatment but this was reversed in the presence of CXCR2 neutralizing antibody (*p*=<0.0012, Kaplan-Meier survival analysis, *n*=8)

CXCR2 was found to be the most upregulated in the genomic analysis with more than 10-fold change. Therefore, using a CXCR2 neutralizing antibody, DCs migration was assessed using the trans-well migration assay. The increased migration seen in sarcosine treated DCs was abrogated when the CXCR2 neutralizing antibody was added to cultured medium (mean percentage of migrated cells: 20.13% for CCL19/21, 35.5%for sarcosine+CCL19/21, 16.33% for sarcosine+CCL19/21 + anti-CXCR2 and 19.46% for CCL19/21 + anti-CXCR2, *p* < 0.0001, one-way ANOVA, *n* = 5) (Fig. 6a & b).

Next, the impact of CXCR2 blockade on anti-tumor efficacy was assessed. B16F10-OVA melanoma tumors were implanted. Animals received infusion of antigen specific OT-I splenocytes and DC vaccines with or without sarcosine. Some animals also received CXCR2 neutralizing antibody one hour before each DC vaccine injection. The animals that were treated with sarcosine treated DCs had significantly slowed tumor growth over time compared to animals receiving non-sarcosine treated DCs and sarcosine treated DCs treated with CXCR2 neutralizing antibody. The mean tumor volume 34 days after tumor implantation was 2926 mm^3^ for controls, 1756 mm^3^ for DC treated mice, 930.7 mm^3^ for sarcosine treated DC treated mice, 1778 mm^3^ for sarcosine treated DCs plus anti-CXCR2 treated mice, and 3111 mm^3^ for anti-CXCR2 treated mice (Fig. 6c). To further evaluated the role of CXCR2 in a relevant intracranial tumor model, GL261-GP100 tumor cells were implanted in mice via an intracranial injection. Animals received antigen specific PMEL splenocytes and DC vaccines. One group received CXCR2 neutralizing antibody one hour before each DC vaccine injection. The animals that were treated with sarcosine treated DCs had significantly prolonged survival compared to animals receiving non-sarcosine treated DCs and sarcosine treated DCs treated with CXCR2 neutralizing antibody. The median survival was 24.5 for control, 27.5 for DC treated mice, 35 for sarcosine treated DC treated mice, 26 for sarcosine treated DCs plus anti-CXCR2 treated mice (Fig. 6d).

### Sarcosine effects on human DC phenotype and migration

**Fig. 7:**
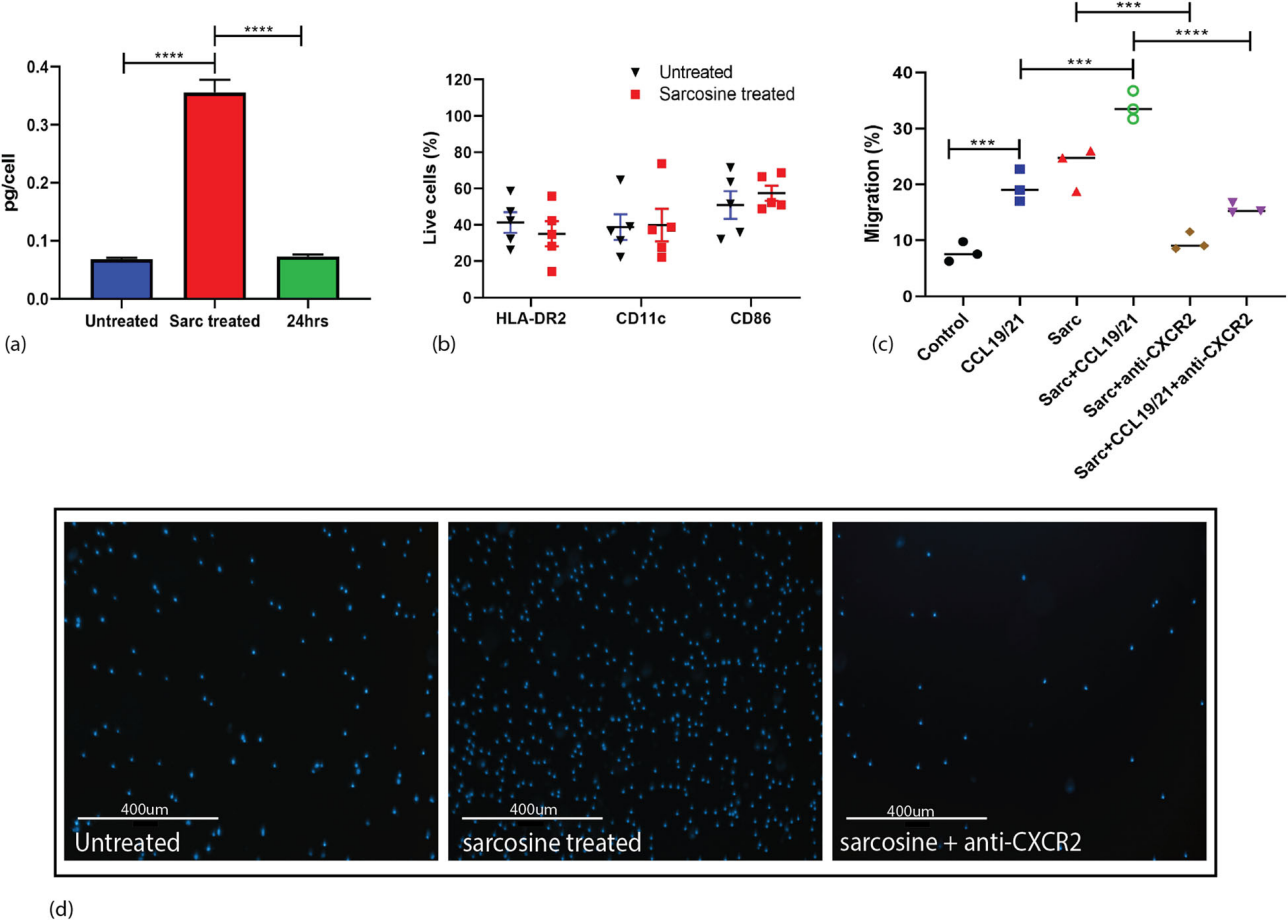
Analysis of human DCs treated with sarcosine in presence of CXCR2 neutralizing antibody (**a**) Mean intracellular sarcosine measurements after sarcosine treatment ranged from 0.064 to 0.4 pg/cell (*p*<0.0001, ANOVA). Human PBMC-derived DCs were treated with sarcosine at 20mM and they were electroporated with CMV pp65-mRNA (antigen). Sarcosine levels were tested after DCs were taken out of sarcosine culture for 24 hours. **b** Flow cytometry of HLA-DR2, CD11c and CD86 in human DCs. **c** In vitro trans-well migration analysis of sarcosine treated-human mDCs demonstrating significant increase in migration of cells with sarcosine. DCs had increased migration with CCL19/21 alone (mean 19.58%) or sarcosine alone (mean 23.17%) compared to the control group (mean 7.833%). Sarcosine and chemokines resulted in increased migrated than either sarcosine or chemokines alone (mean 34.00%). Sarcosine migration effect was abrogated by adding anti-CXCR2 to both sarcosine (mean 9.667%) and sarcosine with CCL19/21 (mean 15.67%) (*P* <0.0001, one-way ANOVA, Human DCs were isolated and pooled from PBMC of five different healthy donor and experiment repeated three times). **d** Immunofluorescent microscopy picture observation of trans-well migration of sarcosine treated human DCs when CXCR2 neutralizing antibody added to the cultured medium. Migrated cells were stained with DAPI. Human DCs were isolated and pooled from PBMC of three different healthy donor and experiment repeated three times

The findings of sarcosine induced-migration was then tested in human DCs. Human PBMC derived DCs were cultured at various concentrations of sarcosine. Human DCs were collected and sarcosine intracellular concentration was measured using chromometric analysis. Sarcosine concentration within cells increased up to 0.4 pg/cell when cells were cultured at 20 mM of sarcosine. Additionally, the intracellular sarcosine returned to control levels when cells were removed from sarcosine containing media within 24 h. Like the murine cells, sarcosine levels could only be transiently increased within the DCs (Fig. 7a). Like murine DCs, sarcosine treatment did not result in changes in HLA-DR2, CD11c and CD86 expression in human DCs compared to control DCs (Fig. 7b). Also, sarcosine treated human DCs showed a significant increase in trans-well migration that was abrogated by adding the CXCR2 neutralizing antibody (mean percentage of migrated cells: 7.833% for control, 19.58% for CCL19/21 alone, 23.17% for sarcosine alone, 34.00% for sarcosine+CCL19/21, 23.17% for sarcosine+Anti-CXCR2 and 15.67% for sarcosine+CCL19/21 + anti-CXCR2, *p* = 0.0026, two-way ANOVA, *n* = 9) (Fig. 7c & d).

## Discussion

DC vaccines are a versatile and potentially potent therapy for treatment resistant tumors such as GBM. Phase I and II studies of DC vaccines for GBM have demonstrated the ability to induce potent adaptive immune responses in patients [6, 13, 14]. We currently have an ongoing phase II clinical trial testing a CMV pp65 RNA DC vaccine for newly diagnosed GBM in which select patients have demonstrated robust immunologic and radiographic responses to treatment (ATTAC II, NCT 02465268). Our prior data has demonstrated that DC vaccine efficacy is predicted by efficient DC migration [6]. Therefore, sarcosine-induced migration has the potential to greatly impact the translation of DC vaccines into an efficacious treatment platform for patients.

Our current data demonstrate a survival benefit of DC vaccines for an intracranial tumor model when sarcosine is added to the DCs. Prior murine studies have only shown a survival benefit when DCs are given prior to tumor implantation or given as an IP injection [15, 16]. The increased DC migration achieved with sarcosine in our studies converted an otherwise non-efficacious platform into a therapy with a survival benefit. Our study is the first description of leveraging sarcosine to increase the migration of immune cells to enhance immunotherapy. Importantly, the doses of sarcosine that used to increase DC migration do not induce tumor invasiveness or growth by itself. In addition, our data demonstrate that sarcosine treated DCs preserve the ability to present antigen and induce T cell proliferation.

These data show that the mechanism of sarcosine enhanced migration is dependent on the upregulation of CXCR2. The findings of CXCR2 upregulation in DCs is a novel finding, although CXCR2 is a known regulator of migration in human immune cells [17]. Human dendritic cells express IL-8 receptors including CXCR1 and CXCR2 and IL-8 can attract dendritic cells through its receptors [18]. CXCR2 expression levels in immature DCs are typically higher than mature DCs [18]. In addition, DCs can secrete IL-8 [19, 20] and CCL5 (RANTES), MIP-la, and MCP-3 [21] chemokines for which CXCR2 is receptor, pointing to possible autocrine function of CXCR2 for DC migration [21]. We have shown that blocking CXCR2 nullifies sarcosine induced DC migration. Sarcosine is known to compete with glycine for the glycine transporter type-1 (Gly-T1) receptor on the cell membrane [22], therefore decreasing intracellular glycine. This reduction results in oxidative stress [23, 24]. This stress likely leads to an increase in arachidonic acid and Cox 1 [25]. Cox 1 upregulates the CXC chemokine family [26] leading to an increase in cell migration [21, 27–30].

Overall, sarcosine is a non-toxic compound that increases DC migration leading to improved outcomes in a tumor model treated with sarcosine treated DC vaccines. Sarcosine has a similar effect on human DCs. Therefore, this strategy could be easily translated into clinical protocols being utilized to treat cancer with DC based immunotherapy. Translational studies are necessary to further evaluate the efficacy of a sarcosine treated DC vaccine strategy in the treatment of brain tumors.

## Conclusion

Sarcosine increases the migration of murine and human DCs via the CXC chemokine pathway. This increase in DC migration also resulted in a more robust anti-tumor immune response and better tumor control and prolonged survival in the intracranial and flank murine tumors models. Sarcosine is non-toxic to murine and human DCs. Further studies in human subjects are necessary to determine the utility of this treatment platform.

## Supplementary information

**Additional file 1: Figure S1.** Measurement of antigen uptake in murine BM-DC and induction of T cell proliferation. **Figure S2.** The efficacy of sarcosine loaded DCs in tumor bearing mice. (**a**) Body weight was measured every ten days post B16F10-ova subcutaneous implantation.

## Abbreviation

APCs: Antigen presenting cells
Cox 1: Cyclooxygenase 1
CXCR2: C-X-C chemokine receptor type 2
DC: Dendritic cell
GBM: Glioblastoma
Gly-T1: Glycine transporter type-1
GM-CSF: Granulocyte macrophage colony stimulating factor
HCV: hepatitis C virus
HIV: Human immunodeficiency virus
IACUC: Institutional Animal Care and Use Committee
LN: Lymph nodes
PBMCs: Peripheral blood mononuclear cells
RT-PCR: Reverse transcription polymerase chain reaction
SQ: Subcutaneous
Tregs: Regulatory T cells
