# Supplementary material for: Sarcosine promotes trafficking of dendritic cells and improves efficacy of anti-tumor dendritic cell vaccines via CXC chemokine family signaling
Source: J Immunother Cancer. 2019 Nov 21;7:321. doi: 10.1186/s40425-019-0809-4 (PMC6873439; doi:10.1186/s40425-019-0809-4)
Supplement: Supplementary file 1 — Additional file 1: Figure S1. Measurement of antigen uptake in murine BM-DC and induction of T cell proliferation. Figure S2. The efficacy of sarcosine loaded DCs in tumor bearing mice. (a) Body weight was measured every ten days post B16F10-ova subcutaneous implantation. [file 40425_2019_809_MOESM1_ESM.docx]

**Supplementary data**


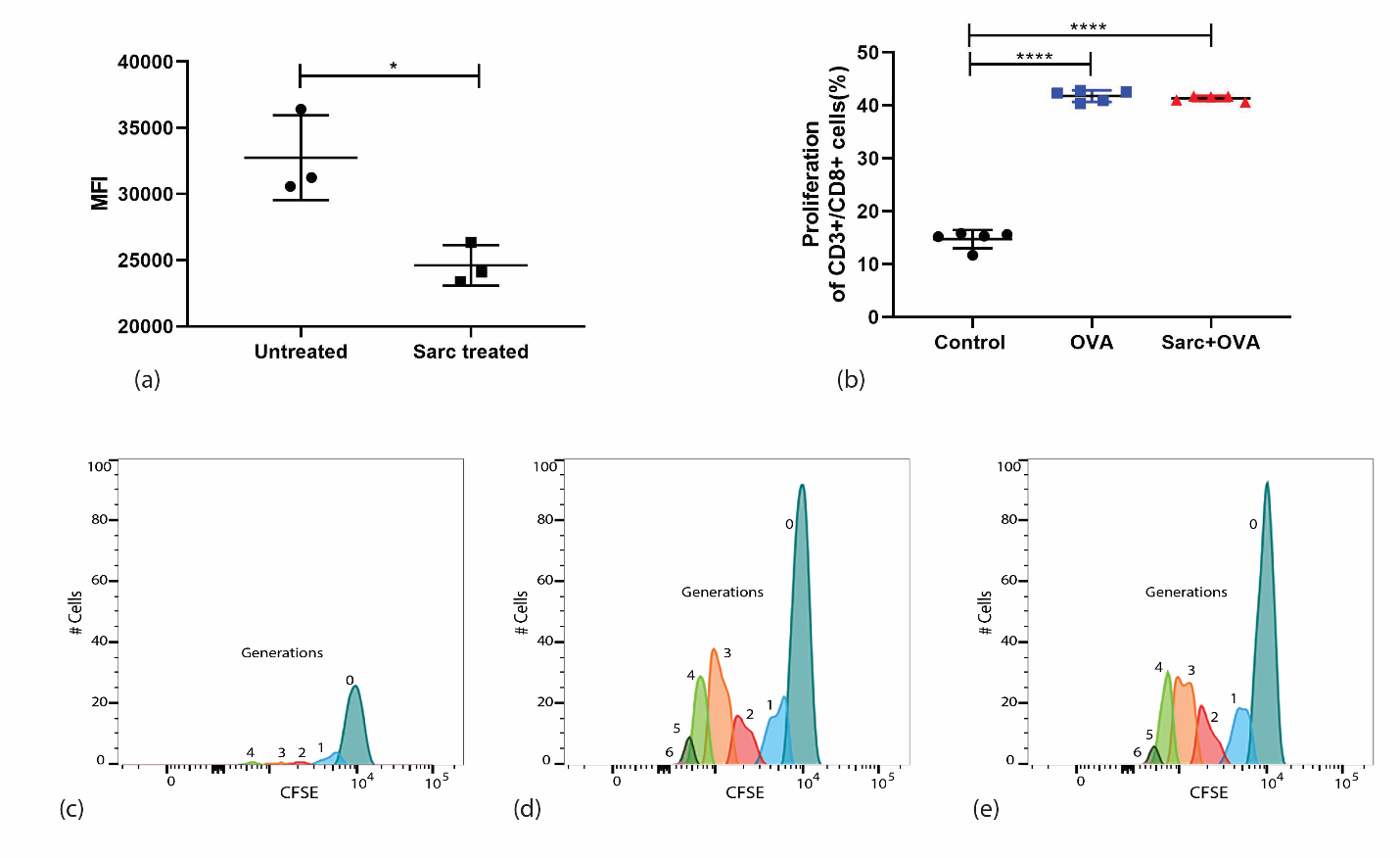


**Supplementary Figure 1.** Measurement of antigen uptake in murine BM-DC and induction of T cell proliferation. (**a**) Cells were incubated with FITC-OVA and antigen uptake was evaluated as mean fluorescence intensity (MFI) of FITC signal. Sarcosine treated DCs had reduced antigen uptake. Mean 32741 MFI for untreated BM-DC vs. 24610 MFI for sarcosine treated BM-DC. (p=0.0165, unpaired t test, n=3). (**b**) In vitro T cell proliferation after co-culture with DCs. Mean 14.72% control BM-DC, 41.76%, untreated BM-DC with OVA-mRNA electroporation and 41.32% sarcosine treated BM-DC with OVA-mRNA electroporation, (n=5). (**c-e**) Representative T cell proliferation following co-culture with control BM-DC, BM-DC electroporated with OVA-mRNA and sarcosine treated BM-DC electroporated with OVA-mRNA.


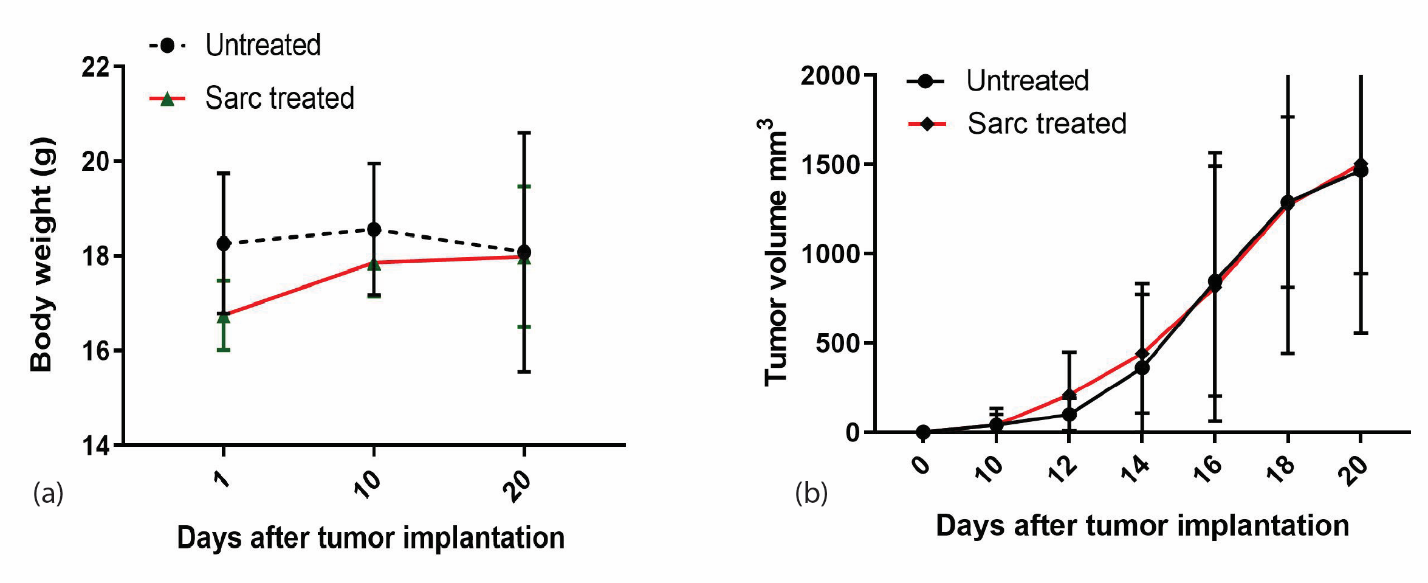


**Supplementary Figure 2.** The efficacy of sarcosine loaded DCs in tumor bearing mice. (**a**) Body weight was measured every ten days post B16F10-ova subcutaneous implantation. Mean 18.21g for DCs and 18.02g for sarcosine loaded DCs at day 20, p= 0.5717, 2 way ANOVA, n=5. (**b**) Sarcosine loaded B16F10-OVA tumor bearing animals versus non-sarcosine treated B16F10-OVA tumor bearing animals. Mean of tumor volume: 1466 mm3 for control and 1504 mm3 for sarcosine loaded B16F10-OVA tumor cells at day 20. (p=<0.9993, 2 way ANOVA, n=10).
